# Rapid reduction in surgical time and high level of accuracy in alignment and femoral component size prediction in robotic‐assisted total knee arthroplasty with ROSA Knee System

**DOI:** 10.1002/jeo2.70148

**Published:** 2025-01-27

**Authors:** Stefano Petrillo, Giorgio Moretti, Niccolò Bordignon, Sergio Romagnoli

**Affiliations:** ^1^ Joint Replacement Department IRCCS Galeazzi‐Sant'Ambrogio Hospital Milan Italy

**Keywords:** knee, learning curve, persona knee system, robotic‐assisted arthroplasty, ROSA, total knee arthroplasty

## Abstract

**Purpose:**

Robotic‐assisted total knee arthroplasty (RA‐TKA) has gained popularity for its potential ability to improve surgical precision and patient outcomes, despite concerns about its long learning curve and increased operative times. The aim of this study is to evaluate the learning curve of the ROSA® Knee System, the relationship between each phase of the learning curve and the accuracy of the robotic system in femoral component size and knee alignment prediction.

**Methods:**

A single surgeon retrospective analysis of total operative time (TOT) and total robotic time was conducted. The first 60 cases of RA‐TKA performed between July 2023 and March 2024 were included. Six (10%) patients were excluded due to incomplete surgical reports. A cumulative sum analysis was used to identify the learning and proficiency phases of the surgeon's learning curve. Moreover, femoral component size prediction accuracy and the difference between planned and achieved knee alignment were analyzed.

**Results:**

The projected learning curve showed a significant reduction in TOT after 10 cases, with mean time decreasing from 62.6 ± 7.92 min in the learning phase to 49.9 ± 8.10 min in the proficiency phase (*p* = 0.0008). The robotic procedure accounted for 48% and 42% of the TOT in the learning and proficiency phases, respectively. Prediction in femoral component size was accurate in 92.6% of cases. The difference between planned and achieved knee alignment was not statistically significant (1.1° ± 0.9°).

**Conclusions:**

The ROSA® Knee System allows a rapid learning curve in RA‐TKA, with a significant reduction in operative time after the first 10 cases. An experienced orthopaedic surgeon specialized in knee arthroplasty can quickly reach a proficiency phase, maintaining high accuracy in alignment and femoral component sizing. These findings suggest that the ROSA® system is an effective and reliable tool for CR RA‐TKA, offering precise and reproducible outcomes.

**Level of Evidence:**

IV.

**Abbreviations**
CR: cruciate retaining
CUSUM: cumulative sum analysis
KET: knee state evaluation time
LFT: landmark femur time
LTT: landmark tibia time
PBT: planning and bone cuts time
RA: robotic‐assisted
TKA: total knee arthroplasty
TOT: total operative time
TRT: total robotic time

## INTRODUCTION

In the past few decades, the number of total knee arthroplasties (TKA) performed each year has kept increasing [12]. However, up to 20% of patients are dissatisfied with the outcome mainly due to pain, instability and malalignment [4, 9, 13]. Robotic‐assisted (RA) TKA rose to popularity with the aim of helping surgeons reduce error rates and, therefore, limit the percentage of unsuccessful TKAs [20, 24, 25]. Several benefits have already been reported with robotic assistance, such as better alignment and knee stability and more precise bone cut orientation [1, 5, 11, 16, 25]. However, the main concerns with RA‐TKA are the increased costs, surgical time and prolonged learning curve compared to the standard manual technique [7, 17, 22].

The understanding of the potential benefits, limits, and learning curve of a novel RA‐TKA system is fundamental in evaluating the impact of its introduction into the surgical workflow. The ROSA® (Robotic Surgical Assistant) Knee System (Zimmer Biomet, Warsaw, Indiana, USA) can be considered a collaborative robotic tool for TKA [2, 10, 14, 15, 18]. The surgeon remains in control of the procedure but collaborates with a robotic system that provides information on knee alignment, stability, femoral component rotation and flexion, femoro‐tibial opening gaps and the dimension of bone cuts. Moreover, the robotic guide suggests an adequate femoral component size in its imageless version and both femoral and tibial component size in its image‐based version [10, 14, 18]. Several studies reported data regarding the learning curve of RA‐TKA with the ROSA Knee System [3, 6, 11, 17, 23]. In all of these, the operative time was estimated as the time between skin incision and wound closure, without considering the total robotic time (TRT) and the specific learning curve for each step of the surgical procedure. Moreover, none of these studies evaluated the learning curve using Persona® CR (Zimmer Biomet, Warsaw, Indiana, USA) TKA, a modern anatomic TKA, and only one author analyzed the experience of a single high‐volume surgeon [11].

In our opinion, in the hands of a high‐volume knee arthroplasty surgeon, ten cases with the ROSA Knee System are enough to achieve a significant reduction in total operative time (TOT).

The aim of our study was to analyze the learning curve of the ROSA Knee System in a cruciate retaining (CR) RA‐TKA, the reliability of femoral component size prediction and the difference between planned and final knee alignment.

## MATERIALS AND METHODS

The present study was approved by the Ethics Committee board of San Raffaele University of Milan. The inclusion criteria were age >18 years old and indication for primary TKA. Exclusion criteria were tumours of the knee, infections, neurological disorders affecting knee function, unstable knee requiring constrained TKA, and TKA in unicompartmental knee arthroplasty revision.

A total of 60 patients who received an RA‐TKA between July 2023 and March 2024 were evaluated retrospectively. Six (10%) patients were excluded from the present study due to incomplete ROSA surgical report. Demographic details of the patients are reported in Table 1.

**Table 1:** Demographic characteristics of the patients.

Patients demographics	Mean ± SD	Range
Age (years)	69.6 ± 8.1	48–84
Sex	12 male 48 female	n.a.
BMI (Kg/m^2^)	26.3 ± 4.5	24–32

Abbreviations: BMI, body mass index; n.a., not applicable; SD, standard deviation.

All surgical procedures were performed between July 2023 and March 2024 by the first author (S. P.), who is a high‐volume knee arthroplasty surgeon (>150 per year) who was not involved in data collection and analysis. The surgeon received 4 h of teaching and 3 h of cadaver‐lab training during a Zimmer Biomet Institute course prior to the first RA‐TKA but has no previous experience with other RA‐TKA systems. On the other hand, he has high experience with the use of PERSONA TKA, performing more than 600 cases in the last 5 years.

### Operative time and femoral component size

The TOT was extracted from the surgical note recorded as the time between skin incision and end of wound closure. Additionally, the following operative time values were extracted from the ROSA‐produced surgical report: TRT, landmark femur time (LFT); landmark tibia time (LTT); knee state evaluation time (KET) and planning + bone cuts time (PBT). The surgical support staff learning curve was not evaluated as the same nurse and anaesthesiologist participated in all the included procedures. The femoral component size was extracted from the surgical report.

### RA‐TKA surgery

**Figure 1:**
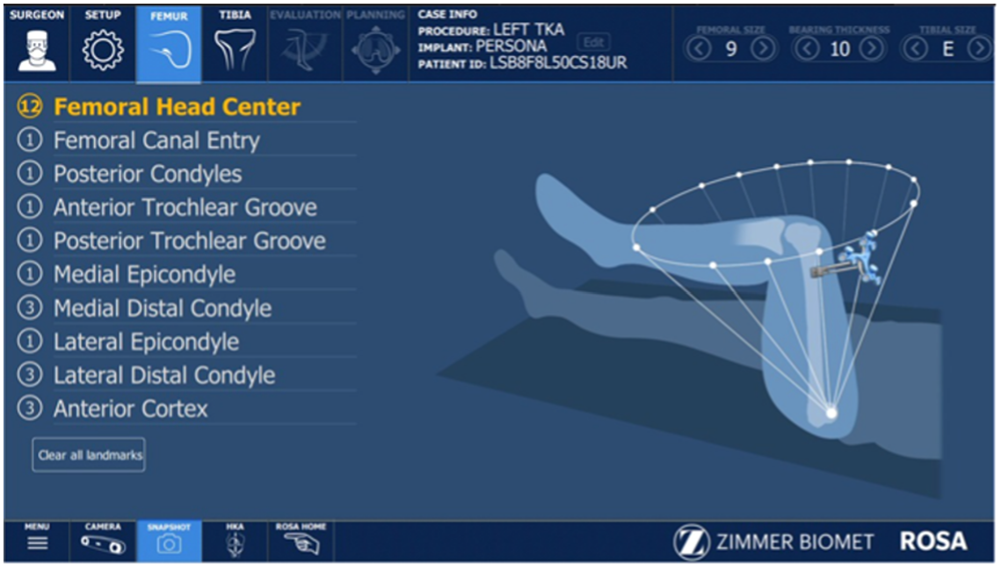
Calibration phase.

**Figure 2:**
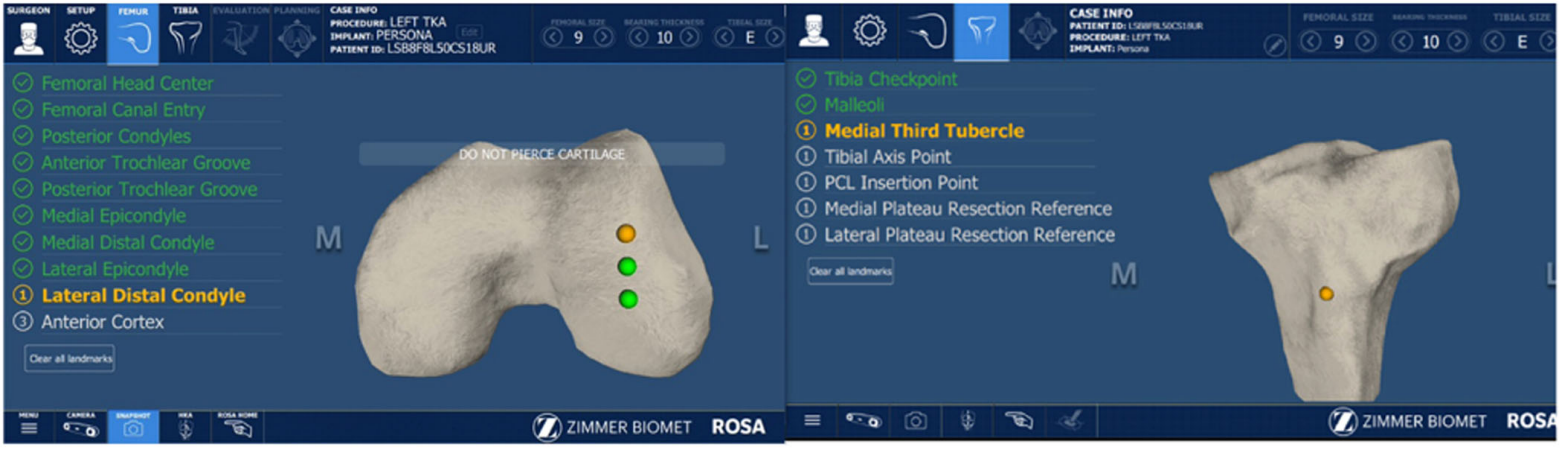
Femoral and tibial landmark acquisition.

**Figure 3:**
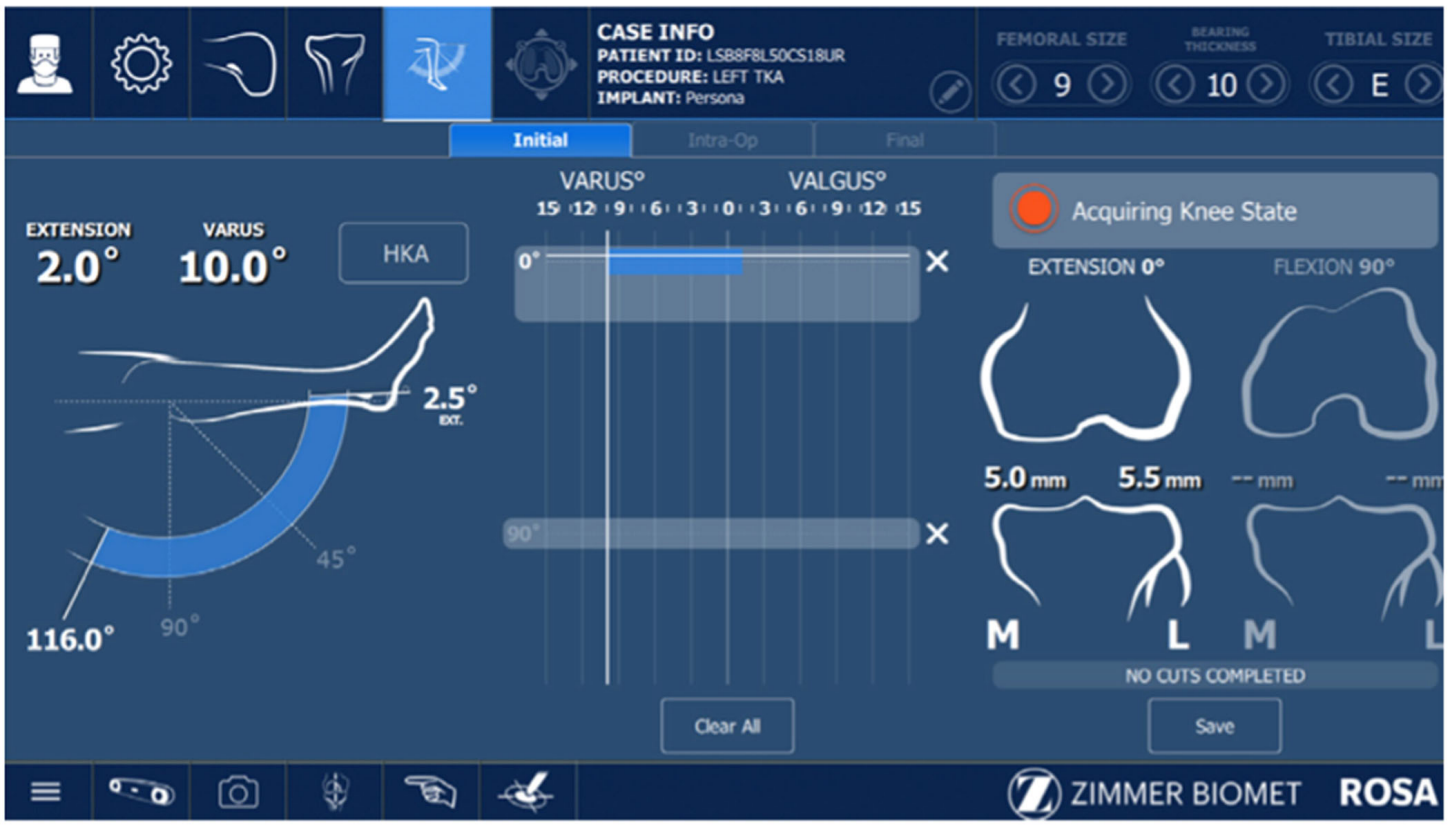
Knee state evaluation.

**Figure 4:**
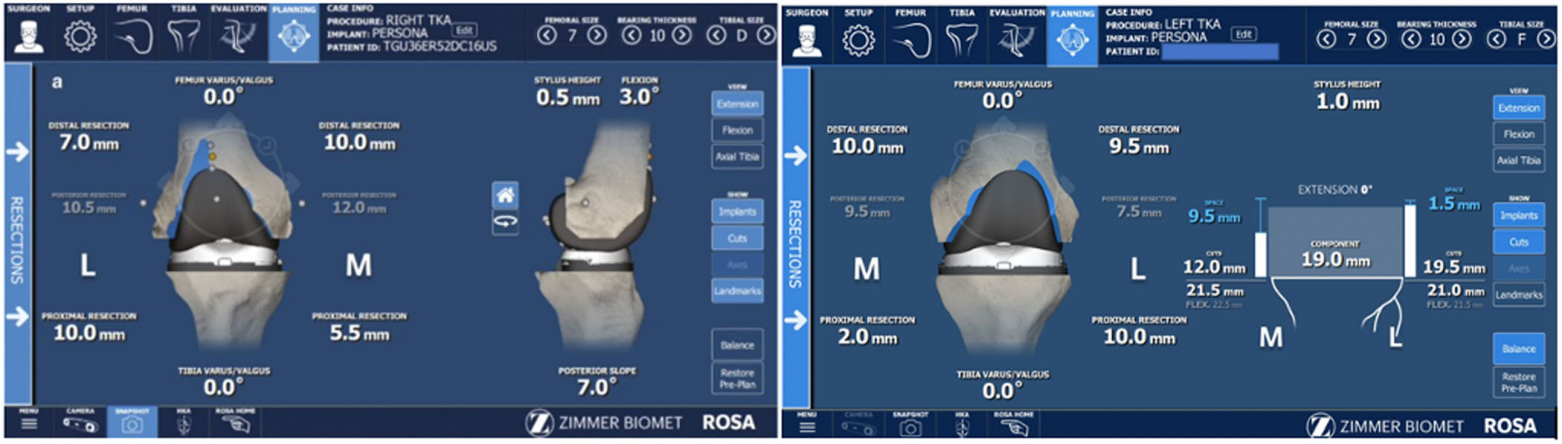
Planning phase.

Every case featured the same operating staff (scrub nurse, second surgeon, resident and the same industry representative). As per the author's preference (S. P.), a restricted kinematic alignment and an adjusted mechanical alignment technique were used in varus and valgus knees, respectively. A mini‐midvastus approach through a single medial parapatellar skin incision was used and a PERSONA fully cemented TKA with a CR femoral component and an ultracongruent polyethylene liner was implanted in all patients. Two femoral pins (3.2 mm diameter) and two tibial pins (3.2 mm diameter) were positioned in the proximal part of the wound and in the distal‐middle third of the medial aspect of the tibia, respectively. Detection of the hip centre of rotation was the first step of the RA‐TKA procedure. In this phase, the surgeon recorded 14 positions of the lower limb in space to calibrate the robotic system (Figure 1). Then, using the ROSA registration pointer, 10 and 7 bony landmarks were acquired in the femur and tibia, respectively (Figure 2). Afterwards, during the KET, the surgeon applied varus/valgus stress in the knee at different degrees of knee flexion in order to evaluate overall knee laxity and stability. The ROSA System displayed and stored data regarding range of motion, varus/valgus deformity and ligament laxity (Figure 3). In the PBT phase, the femur and tibia bone cuts were planned and performed in a collaborative fashion. For each modification set, the ROSA Knee System gave updated information regarding bone resections, angles, extension and flexion gaps and tibial and femoral component size prediction and notified the user about possible complications like notching, out‐of‐range values and implant incompatibility (Figure 4). Then, according to the intraoperative planned values, the ROSA robotic cutting guide moved following the knee in space to execute the tibial and distal femoral cuts and to position the 4‐in‐1 cutting guide in the correct position. The procedure ends with the final implant cementation, insertion of the bearing component trial and the knee state evaluation test using the ROSA software, applying varus/valgus stress at different degrees of knee flexion.

### Statistical analysis

In order to identify the learning effect and the turning point from the learning phase to the proficiency phase, a cumulative sum analysis (CUSUM) was performed. The analyses were conducted on both the TRT and the TOT. Due to the non‐normal distribution, for the comparisons between groups, the Wilcoxon signed‐rank test was used. Variables with normal distribution were reported as mean value ± standard deviation (min–max). Statistical analyses were performed using the SAS® Version 9.4 software.

## RESULTS

**Figure 5:**
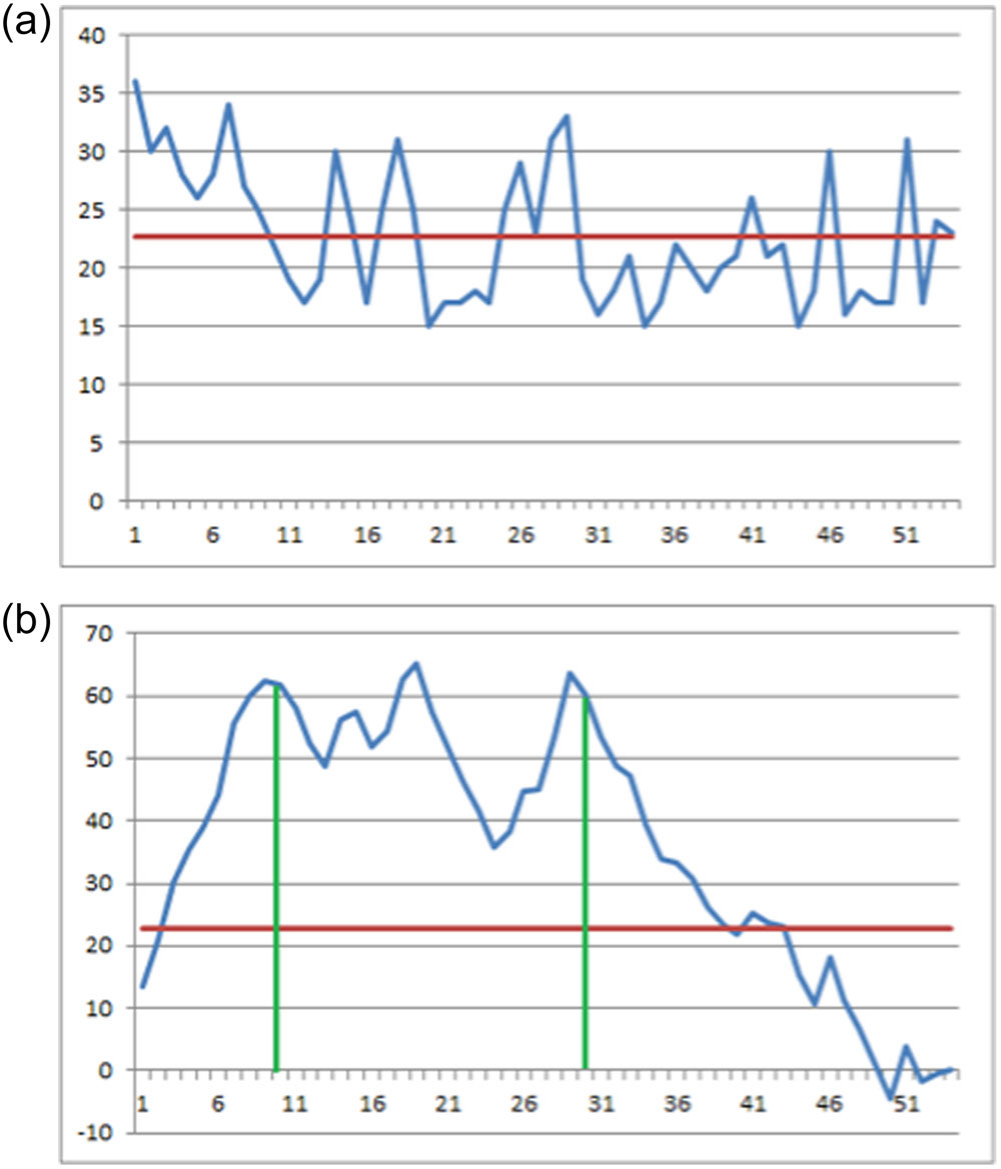
(a) Total robotic time (TRT) – Absolute value plot. (b) TRT – CUSUM plot.

The single TRT in relation to the relevant average was plotted (Figure 5a). The CUSUM analysis (Figure 5b) identified three phases: learning phase I (cases 1–10), learning phase II (cases 11–30) and proficiency phase (cases 31–54).

**Figure 6:**
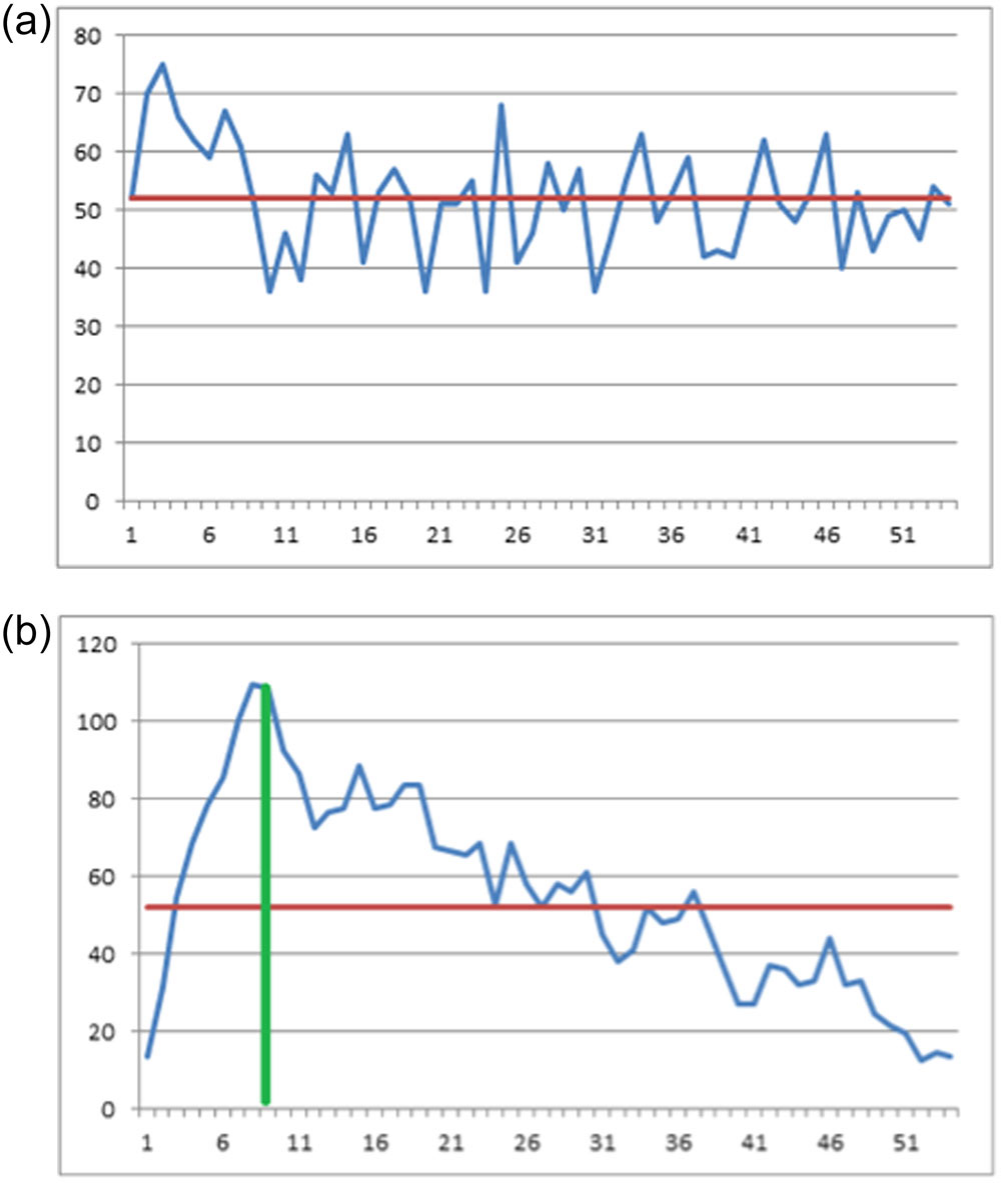
(a) Total operation time (TOT) – Absolute value plot. (b) Total operation time (TOT) – CUSUM plot.

The total observations were divided into three groups, and the groups of phase 1 and phase 3 were compared in terms of surgical duration. The mean values of phase 1 and phase 3 groups were 28.8 ± 4.26 min (10–36) and 20.1 ± 4.31 min (14–21), respectively (*p* > 0.0001) (Table 2). Then the procedure was repeated for the TOT, and the CUSUM analysis identified two phases: learning phase (cases 1–10) and proficiency phase (cases 11–54) (Figure 6a,b). The mean operative time of the learning phase was 62.6 ± 7.92 min (51–75), while it was 49.9 ± 8.10 min (36–63) in the proficiency phase (*p* = 0.0008) (Table 3).

**Table 2:** Total robotic time by group.

Parameter	Learning phase I	Proficiency phase	*p* value
Cases	10	24	
Mean ± SD (min)	28.8 ± 4.26	20.1 ± 4.31	
Median	28.0	19.0	<0.0001
Min–Max	10–36	14–21	
95% CI	25.8; 31.8	18.3; 21.9	

**Table 3:** Total operation time (TOT) by group.

Parameter	Learning phase	Proficiency phase	*p* value
Cases	10	44	
Mean ± SD (min)	62.6 ± 7.92	49.9 ± 8.10	
Median	62.0	51.0	0.0008
Min–Max	51–75	36–68	
95% CI	56.5; 68.6	47.4; 52.3	

The mean TOT was 51.9 ± 9.3 min (36–75), while the mean TRT was 22.6 ± 5.7 min (15–36 min), indicating that the robotic procedure represents 42% of the entire surgery duration. The mean LFT, LTT, KET and PBT were 2.3 ± 0.6 min (2–4), 1.1 ± 0.3 min (1–3), 4.1 ± 2.8 min (1–14) and 14.3 ± 4.4 (4–24), respectively. PBT represented 65% of TRT, while LFT, LTT and KET resulted in 10%, 5% and 20% of TRT, respectively.

The femoral component size prediction was accurate in 50 (92.6%) and failed in 1 and 2 sizes in 3 (5.6%) and 1 (1.8%) patients, respectively.

The difference between planned (with ROSA software) and achieved (measured with full‐length weight‐bearing anteroposterior lower limb X‐ray) HKA was 1.1° ± 0.9° (0–3; *p* = 0.6).

No patients experienced medical or implant‐related complications in the early postoperative days after surgery and at a mean follow‐up of 9.3 ± 2.4 months (6–13).

## DISCUSSION

The main finding of this study is that, in the hands of a high‐volume knee arthroplasty surgeon, a rapid reduction in operative time with a transition from learning to proficiency phase of RA‐TKA can be obtained following only 10 cases using the ROSA Knee System. A significantly accurate prediction was observed comparing planned and achieved mechanical axis alignment. Moreover, the difference between planned and final femoral component size was not significant, and no differences were found in 92.6% of cases. These results highlighted that, with careful registration of the robotic system, and following a sufficient training period, excellent and reproducible results can be obtained in RA‐TKA with ROSA Knee System.

Other authors have analyzed the learning curve of the same robotic assistant device. Dragosloveanu et al. [6] were the first to report the experience of 3 different surgeons performing 13 consecutive RA‐TKA each in a period of 6 months using a posterior stabilized (PS) TKA. Their mean operating time was 111.54 min in the learning phase, decreasing to 86.43 after 6, 3 and 4 cases for the 3 surgeons. Similar results were found by Bolam et al. [3] who analyzed the learning curve of 53 RA‐TKA using both CR and PS implants but without specifying the exact number of each implant in a period of 21 months performed by 3 different surgeons. Their mean operative time was 114 and 110 min in the learning and proficiency phase, respectively. For only one surgeon, the learning phase ended after case 15. Vanlommel et al. [23] reported the experience of 3 different surgeons performing their first 30 RA‐TKA in a period of 9 months. They found a decrease in the mean operative time from 110 min of the learning phase to 86 min of the proficiency phase after 6, 10 and 11 cases for the 3 surgeons involved. Recently, Kenanidis et al. [11] investigated the number of RA‐TKA cases necessary for a single surgeon to match the operative time of conventional manual TKA with robotic TKA. They stated that 73 RA cases were necessary to reach a mean operative time of 80 min, which was similar to manual TKA time.

In this study, we also evaluated the impact of robotic procedure steps on TOT. According to our results, 48% of the surgical procedure was robotic in the learning phase, decreasing to 42% in the proficiency phase. These results suggest that, after an initial learning of only 10 cases, the robotic time has no significant impact on TOT. In particular, PBT was 59% and 64% of TRT in the learning and proficiency phase, respectively, demonstrating that the surgeon became more confident with the landmarking and knee stability check, but a significant reduction of surgical time, including planning and bone cuts, remains difficult to obtain.

We believe that our results are different from those reported in the literature because of 3 main reasons. First, we implanted only CR‐TKA, while in the majority of the previous studies, a PS implant was used: it is well known that PS implants require additional surgical acts for femur preparation, resulting in increased operative time. Second, we reported the experience of a single high‐volume knee arthroplasty surgeon who had no previous experience with other navigation or robotic systems but who had cadaver‐lab training prior to the first use of the ROSA Knee System and also had a high frequency of use of the robot and PERSONA TKA. Indeed, 67 consecutive RA‐TKA with the same implant were performed in 7 months using the same surgical approach and standardized technique. On the other hand, previous studies have reported the cumulative experience of different surgeons performing all together 13, 53 and 30 cases in 6, 21 and 9 months, respectively [3, 6, 23]. Third, every RA‐TKA was performed not only by the same surgeon but also by the same surgical staff (scrub nurse, surgical assistant, anaesthesiologist, biomedical engineer). In this manner, the operating room environment and the entire procedure were standardized, resulting in a more efficient and less time‐consuming overall process. However, when considering alignment accuracy and femoral component size prediction, our results are similar to those reported in previous studies, with no significant difference in the percentage of 3° outliers for HKA [19]. Moreover, in only 7.4% of the patients, an error in femoral component size prediction was noticed, and all of them were oversized.

Several factors influence the learning curve of a novel robotic system [8, 21]. Surgeons with more experience in orthopaedic surgery and in using robotic technologies, navigation and computer devices generally have a faster learning curve. Another important aspect is surgeon training: in this particular case, the ROSA Knee System manufacturer not only gave theoretical lessons, but they also provided cadaver‐lab training and surgeon to surgeon visits. Furthermore, intensive online courses, as well as hands‐on workshops offered by Zimmer Biomet Institute, could have accelerated the learning process, resulting in reduced operative time and reproducibility of the RA surgery. Moreover, continuous support from the biomedical engineers during the early stages in the operating room could help to resolve concerns and improve the surgeon's confidence in using the system, reducing the risk of perioperative complications as well as malfunctioning of the robotic system. Finally, as shown in our study, the more frequently the surgeon performs RA‐TKA, the more the surgeon quickly becomes familiar with robotic features and advantages, quickening the procedure and limiting complications. Finally, consistent practice helps develop specific skills and improve the precision and efficiency of surgical procedures.

The main strength of the present study was that all RA‐TKA were performed by the same high‐volume knee arthroplasty surgeon in a short period of time. Moreover, data were extracted from medical reports and ROSA computer by an independent investigator, who was not involved in the surgical procedure. Another strength is that the same surgical staff participated in all the surgical procedure evaluated. Furthermore, we have included patients with knee OA with severe varus/valgus deformities, fracture or osteotomies sequalae, making the sample of cases included as varied as possible.

Limitations of the present investigation must be underlined: retrospective design of the study, absence of control group of patients managed with conventional manual TKA, short duration of follow‐up and no clinical and functional evaluation of outcomes.

## CONCLUSIONS

The TOT of RA‐TKA using ROSA significantly decreased after 10 cases. High accuracy was observed between planned and achieved mechanical axis alignment. Moreover, the difference between planned and implanted femoral component size was not significant.

The impact of robotic time on TOT was 48% in the learning phase and 42% in the proficiency phase. The PBT remains the core phase of the procedure and was around 60% of the TOT in both the learning and proficiency phases.

## AUTHOR CONTRIBUTIONS

**Stefano Petrillo**: Conceptualization; methodology; investigation; writing—original draft preparation; writing—review and editing. **Giorgio Moretti**: Methodology; investigation; writing—original draft preparation; writing—review and editing. **Niccolò Bordignon**: Methodology; investigation; writing—review and editing. **Sergio Romagnoli**: Conceptualization; investigation; supervision. All authors have read and agreed to the published version of the manuscript.

## CONFLICT OF INTEREST STATEMENT

Sergio Romagnoli is a paid consultant for Zimmer Biomet. Stefano Petrillo is a paid consultant in medical education for Zimmer Biomet. The remaining authors declare no conflicts of interest.

## ETHICS STATEMENT

This protocol will be conducted in accordance with ethical principles originating from the Declaration of Helsinki, in compliance with Good Clinical Practice and applicable regulatory provisions. The Ethics Committee expresses a favourable opinion and requests that the information indicated by the Bioethicist be included in the Informed Consent. IRB: ALLCCP, Em. 225–2024.

## Data Availability

The original contributions presented in the study are included in the article/Supplementary Material, and further inquiries can be directed to the corresponding author.
